# MMP Inhibitors and Dentin Bonding: Systematic Review and Meta-Analysis

**DOI:** 10.1155/2021/9949699

**Published:** 2021-05-27

**Authors:** O. Kiuru, J. Sinervo, H. Vähänikkilä, V. Anttonen, L. Tjäderhane

**Affiliations:** ^1^Research Unit of Oral Health Sciences, Department of Cariology,Endodontology and Paediatric Dentistry, University of Oulu, Oulu, Finland; ^2^Infrastructure of Population Studies, Faculty of Medicine, University of Oulu, Oulu, Finland; ^3^Medical Research Centre, University of Oulu and Oulu University Hospital, Oulu, Finland; ^4^Department of Oral and Maxillofacial Diseases, University of Helsinki,and Helsinki University Hospital, Helsinki, Finland

## Abstract

**Objectives:**

Resin-dentin bond strength decreases over time. This reduction is related to the loss of hybrid layer integrity. Collagenolytic enzymes, especially matrix metalloproteinases (MMPs), are responsible for the degradation of the collagen matrix of the hybrid layer. Various MMP inhibitors with the ability to prevent enzymatic degradation have been identified. This study aimed to systematically review the literature for studies which evaluated the effect of MMP inhibitors on the immediate and aged dentin bond strengths. *Study Selection*Screening and analysis were carried out by two reviewers. Two databases were searched, and from a total of 740 articles, 43 were accepted for full review. 21 articles with 0.2%–2% chlorhexidine (CHX) treatments were included for meta-analysis. A risk of bias assessment was performed on all studies chosen for meta-analysis. A variety of MMP inhibitors have been studied, CHX being the most widely used.

**Conclusions:**

A clear trend for a lower loss of dentin bond strength was observed with different MMP inhibitors. In meta-analysis, no significant difference was seen between the CHX and control in the immediate bond strengths. Bond strengths in the CHX group were significantly higher than the control group after aging (*P* < 0.001). The percentage of fractures occurring at the adhesive interface increased after aging. Five out of 21 studies included in the meta-analysis had high and the rest medium risk of bias. More long-term studies with lower risks of bias should be carried out to increase the reliability of results. *Clinical Relevance*The use of MMP inhibition with chlorhexidine can be recommended to increase the longevity of resin-dentin bond strength.

## 1. Introduction

Studies have shown that the bond between the adhesive systems and dentin weakens over time. This decrease in bond strength is related to the degradation of the hybrid layer [1, 2]. The hybrid layer is the area of adhesion formed by the dentin collagen matrix and resin adhesive. After exposure to acid (etch-and-rinse adhesives) or acidic monomers (self-etch adhesives), the demineralized dentin collagen matrix is infiltrated with the applied adhesive resin [3, 4]. The collagen matrix is vulnerable to enzymatic degradation by the endogenous collagenolytic enzymes, matrix metalloproteinases (MMPs) and cysteine cathepsins, and these enzymes play an important role in bond destruction [2]. MMP inhibitors can prevent the function of these enzymes, with chlorhexidine (CHX), galardin, and benzalkonium chloride being the most widely studied [5, 6]. More recently, collagen cross-linker agents have also been shown to inhibit protease enzymes [7].

Systematic review and meta-analysis by Montagner et al. [8] indicated that CHX is effective in maintaining long-term bond strength. This study aimed to provide an updated systematic review and meta-analysis of the literature involving studies which evaluated the effect of MMP inhibitors on the immediate and aged resin-dentin bond strength. The hypothesis was that MMP inhibitors have a significant effect on the longevity of bond strength, which becomes evident after 6 months.

## 2. Materials and Methods

In this systematic literature review, two separate electronic databases were used (PubMed and Scopus). Suitable search phrases for both databases were constructed using the following search phrases as a guideline: (matrix metalloproteinase*∗* OR MMPs inhibitor*∗* OR protease inhibitor*∗* OR chlorhexidine*∗* OR benzalkonium chloride*∗* OR BAC*∗*) AND (dentin*∗* adhesive*∗* OR adhesive system*∗* OR hybrid layer*∗* OR bond*∗*) AND (ag*∗* OR stability*∗* OR durability*∗* OR strength*∗* OR long-term*∗*) and “cross-linker OR cross-linker”: e.g., with the following search phrases “grape seed extract AND dentin AND bonding” or “proanthocyanidin AND dentin AND bonding.”

The search phrases for each database were altered and optimized so that the resulting articles were as relevant as possible to the targeted articles. The final search phrases used were as follows:

PubMed: (((((((((((proanthocyanidin AND dentin AND bonding) OR (grape seed extract AND dentin AND bonding) OR chlorhexidine*∗* [Text Word] OR “benzalkonium chloride*∗*” [Text Word] OR BAC*∗* [Text Word] OR “matrix metalloproteinase inhibitor*∗*” [Text Word] OR “MMP*∗* inhibitor*∗*” [Text Word] OR “protease inhibitor*∗*” [Text Word]))) AND ((ag*∗* [Text Word] OR stability*∗* [Text Word] OR durab*∗* [Text Word] OR strength*∗* [Text Word] OR long-term*∗* [Text Word]))) AND ((dentin*∗* AND adhesive*∗* [Text Word] OR adhesive system*∗*” [Text Word] OR “hybrid layer*∗*” [Text Word] OR bond*∗* [Text Word])))) OR (((“matrix metalloproteinase*∗*” [Text Word]) AND ((ag*∗* [Text Word] OR stability*∗* [Text Word] OR durab*∗* [Text Word] OR strength*∗* [Text Word] OR long-term*∗* [Text Word]))) AND ((dentin*∗* AND adhesive*∗* [Text Word] OR “adhesive system*∗*” [Text Word] OR “hybrid layer*∗*” [Text Word] OR bond*∗* [Text Word]))))) OR (((((“matrix metalloproteinase inhibitor*∗*” [Text Word] OR “MMP*∗* inhibitor*∗*” [Text Word])) AND bond strength[Text Word])) OR ((“Matrix Metalloproteinase Inhibitors”[Mesh]) AND (((“Dental Bonding”[Mesh: noexp]) OR “Light-Curing of Dental Adhesives”[Mesh]) OR “Self-Curing of Dental Resins”[Mesh])))).

Scopus: (TITLE-ABS-KEY ((proanthocyanidin AND dentin AND bonding) OR (grape seed extract AND dentin AND bonding) OR “matrix metalloproteinase*∗*” OR “MMP*∗* inhibitor*∗*” OR “protease inhibitor*∗*” OR chlorhexidine*∗* OR “benzalkonium chloride*∗*” OR bac*∗*) AND TITLE-ABS-KEY (adhe*∗* OR “hybrid layer*∗*” OR bond*∗*) AND TITLE-ABS-KEY (ag*∗* OR stability*∗* OR durab*∗* OR strength*∗* OR long-term*∗*) AND TITLE-ABS-KEY (dentin*∗*)) AND NOT INDEX (medline) AND (LIMIT-TO (SUBJAREA, “DENT”)).

Using these search phrases, 531 articles were found on PubMed and 209 articles on Scopus. The search included all articles published before 5.7.2018. After the database search, screening was performed by two individuals on all articles to single out the relevant ones. For the screening, the following predetermined rejection criteria were used: under 6 months aging (follow-up), thermocycling used for aging, no measured data of bond strength, no MMP inhibitors used during bonding, no control group, review articles, or other interests, e.g., root canal sealers and root canal posts. If any one of these factors was present, the article was rejected. The article also had to be written in English.

**Figure 1:**
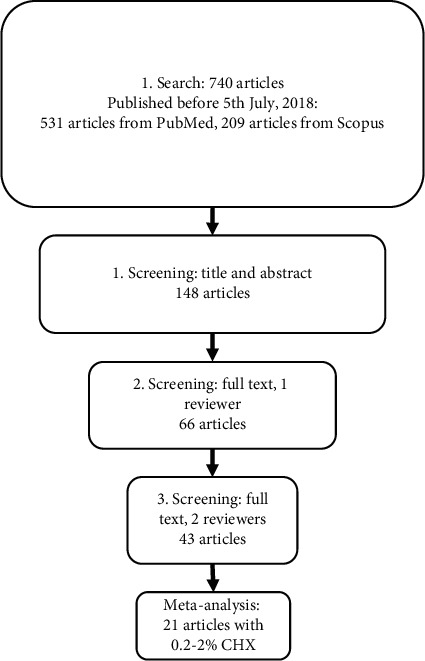
Protocol for the systematic literature review.

The screening was performed in three stages (Figure 1). During the first stage, only the title and the abstract of the article were used to determine whether any of the rejection criteria was present. The initial screening resulted in 126 articles from PubMed and 22 articles from Scopus. During the second stage, the complete text of all remaining articles was read and interpreted by the individual screeners (a total of 148). The same rejection criteria were used, and after careful selection, 59 articles from PubMed and seven articles from Scopus were accepted (*n* = 66). A final screening was performed with both reviewers present. Five of the seven articles found in Scopus were the same as on PubMed. Furthermore, 18 studies were rejected due to the rejection criteria and lack of available data, leaving 43 accepted articles. In addition, studies done on carious teeth were excluded.

Due to the wide heterogeneity between the studies using other MMP inhibitors than CHX, only data involving 0.2–2% CHX would be used for the meta-analysis. The 21 chosen articles were allocated into six different comparison groups depending on whether the samples had been aged for 6, 12, or 24 months and whether an etch-and-rinse or self-etching system had been used. No articles were found for the 12-month storage time and self-etch, so this group was excluded. The bond strength, sample size (*N*), and standard deviation data for the respective storage times and controls were retrieved from the articles. The *N* for each group represented the total number of teeth used in each comparison group. Pooled effect estimates were attained by comparing the means of each bond strength value, expressed as the raw mean difference among the groups. Statistical heterogeneity of the treatment effect was assessed via the Cochran *Q* test, with *P* < 0.05 considered significant, and the inconsistency *I*^2^ test, in which values > 50% were considered to indicate high heterogeneity. Meta-analysis on the chosen comparison groups was carried out using the MedCalc (version 19.2.1: MedCalc Software Ltd., Ostend, Belgium).

### 2.1. Assessment of Risk of Bias

The risk of bias evaluation, adapted from a previous study [8], evaluated the following parameters for the study's quality assessment: randomization, use of intact teeth, use of materials according to the instructions, adhesive procedures performed by the same operator, description of sample size calculation, and blinding of testing. The articles reporting 5 to 6 items were classified as low risk of bias, 3 or 4 as medium risk, and only 1 or 2 as high risk.

## 3. Results

From the initial 740 articles, 43 articles with altogether 240 groups were subjected to a comprehensive examination (Table 1). Altogether, 21 different enzyme inhibitors were tested. The most commonly used MMP inhibitor was CHX (32 studies), followed by BAC (seven studies). Artificial saliva and distilled water were the most commonly used modes of storage for aging. Percentage decreases in bond strength were calculated, and a general trend in the decrease of bond strength with time could be observed. A clear trend for the lower decrease in bond strength with MMP inhibitors in 35 out of 43 studies was also observed, with 13 different enzyme inhibitors showing significantly (at least 50%) lower percentage loss of bond strength compared to the respective control group. The adhesive-mixed fracture percentages for the control and CHX groups ranged from 0 to 100 at baseline, from 41 to 100 at 6 months, from 50 to 100 for the control, and from 55 to 100 for CHX at 12 months, and from 77 to 100 for the control and from 75 to 100 for CHX in the final aging group of 24 months.

### 3.1. Meta-Analysis

**Figure 2:**
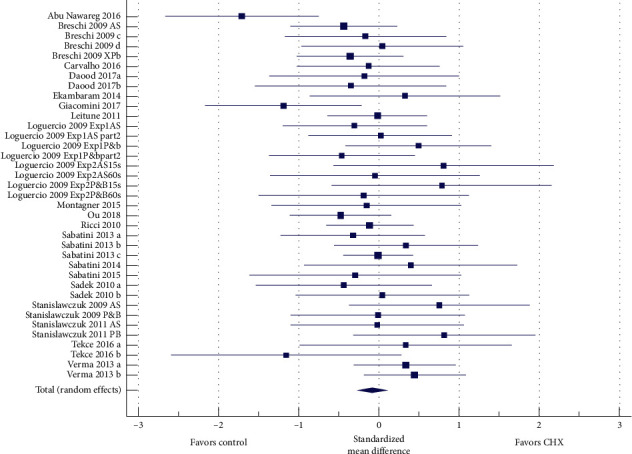
Forest plot of studies at baseline. The *N* for both groups was 396 samples. The total random effect standardized mean difference (SMD) was −0.0821 (CI 95% −0.240; 0.076). The difference was not statistically significant (*t* = −1.019, *P*=0.308). The *I*^2^ (inconsistency) was 21.68%.

A total of 21 articles were subjected to meta-analysis. The first analysis involved CHX vs. control at baseline (Figure 2), including 37 data sets from 21 articles. No significant difference in bond strength was present between the groups (*P*=0.308). The heterogeneity between the studies was low (Cochran's *QP* > 0.05, *I*^2^ 21.7%).

**Figure 3:**
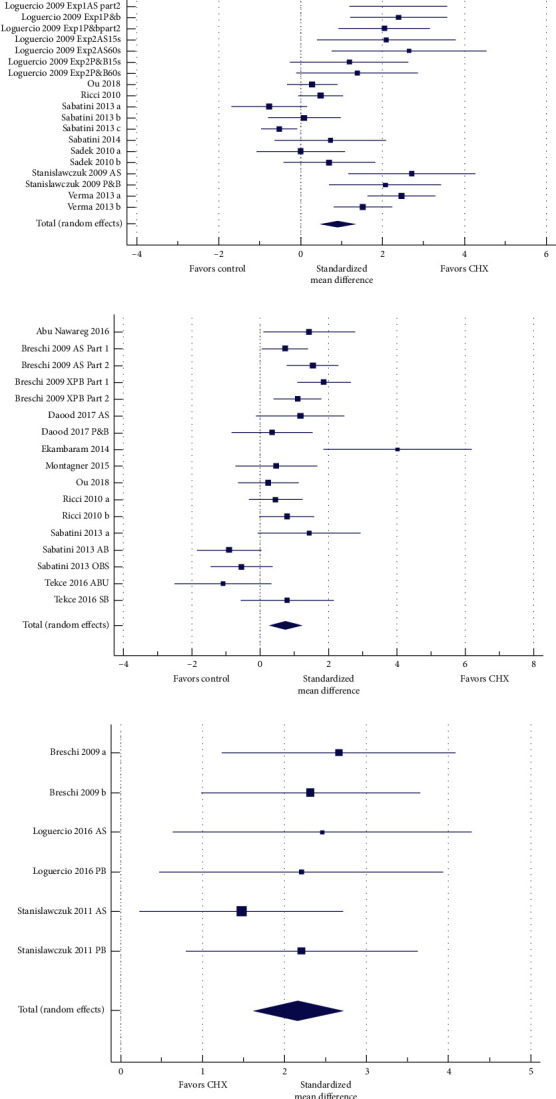
Forest plots of studies after 6, 12, and 24 months of aging. (a) Chlorhexidine (CHX) vs. control at six months. The *N* for both groups was 339 samples. The total random effect SMD was 0.907 (CI 95% 0.517; 1.297). The difference was statistically significant (*t* = 4.568, *P* < 0.001). The *I*^2^ was 82.11%. (b) CHX vs. control after 12 month aging. The *N* for both groups was 173 samples. The total random effect SMD was 0.821 (CI 95% 0.367; 1.275). The difference was statistically significant (*t* = 3.557, *P* < 0.001). The *I*^2^ was 74.97%. (c) CHX vs. control after 24-month aging. The *N* for both groups was 40 samples. The total random effect SMD was 2.168 (CI 95% 1.627; 2.708). The difference was statistically significant (*t* = 7.982, *P* < 0.001). The *I*^2^ was 0.00%.

Thirty data sets from 16 articles were available for the comparison between CHX vs. control after 6 months aging (Figure 3(a)). Bond strengths in the CHX group were significantly higher than in the control group after aging (*P* < 0.001). The heterogeneity between the studies was high (Cochran's *QP* < 0.05, *I*^2^ 82.1%).

To compare CHX vs. control after 12 months aging, 17 data sets from 10 articles were available (Figure 3(b)). Bond strengths in the CHX group were significantly higher than in the control group (*P* < 0.001). The heterogeneity between the studies was high (Cochran's *QP* < 0.05, *I*^2^ 75.0%).

The last analysis involved CHX vs. control after 24 months aging, including six data sets from three articles (Figure 3(c)). Bond strengths in the CHX group were significantly higher than in the control group (*P* < 0.001). The heterogeneity between the studies was extremely low (Cochran's *QP*=0.817, *I*^2^ 0%).

### 3.2. Risk of Bias

Of the 21 articles selected for the meta-analysis, five were classified as having a high risk and 16 as a medium risk of bias. None of the articles had a low risk of bias (Table 2).

## 4. Discussion

The general trend of all 43 articles with 21 different collagenolytic enzyme inhibitor protocols demonstrated markedly lower loss of bond strength with enzyme inhibition. Studies involving other MMP inhibitors were excluded from the meta-analysis due to a wide range of heterogeneity in the inhibitors used and a small number of studies for each inhibitor except for CHX. All three comparisons between the CHX-treated and controls after aging for at least six months demonstrated significantly higher bond strength with CHX. Thus, the hypothesis was accepted.

The results of the meta-analysis showed that the use of CHX has no significant effect on immediate resin-dentin bond strength. The finding is in line with a previous study [8]. Two studies [14, 23] have shown a significant decrease in the immediate bond strength of the CHX group compared to the control. After reviewing the articles, no clear explanation for this difference could be identified, although several differences in the application of CHX were noted. Giacomini and co-authors speculated that the use of acidic CHX after acid etching may have resulted in increased collagen exposure, possibly reducing the immediate bond strength [14].

After aging for 6, 12, and 24 months, the meta-analysis demonstrated significantly better bond strength with CHX compared to the control groups. Despite the heterogeneity of the studies, 6- and 12-month analyses indicate the advantage of using CHX to preserve the bond strength. In addition, with the studies evaluating the bond strength after 24 months of aging, the homogeneity of the data was striking (*I*^2^ 0%). Indeed, longer aging seems to increase the difference between the bond strengths of CHX and control groups.

The immediate fracture percentage at the adhesive interface was practically the same for MMP inhibitor and control groups, although some isolated differences can be identified. It can generally be observed that as the follow-up time increases, the percentage of fractures occurring at the adhesive interface seems to increase, regardless of enzyme inhibition. This may be due to slow degradation of hybrid layer collagen despite the enzyme inhibition, the hydrolytic degradation of the resin component, or—most likely—to both [2, 6]. This supports the idea that bond strength decreases over time and that the adhesive interface plays a significant role in the mode of fracture.

A thorough risk of bias assessment was also carried out to identify the main factors which could affect the creditability of the findings. Five articles were classified to have a high risk of bias, and none with low risk. The results are in line with the respective previous study [8]. None of the studies mentioned sample size calculations, and all but one failed to mention the blinding of the operator performing the bond strength testing. The results may reflect the standard level of reporting of bond strength studies, but at least the blinding of the person performing the bond strength testing should be done and also reported.

## 5. Conclusions

This systematic review and meta-analysis demonstrated that studies strongly indicate the benefits of collagen-degrading enzyme inhibition on the preservation of dentin bond strength. Since CHX does not have any adverse effects on the immediate bond strength, the clinical use of CHX can be recommended to increase the longevity of resin-dentin bonds.

## Figures and Tables

**Table 1:** Percentages of reduction in bond strength during the follow-up period.

Article	Adhesive/mixed failure modes (%) in groups immediately/after aging	*N*	Technique of bonding (E&R/SE) MMP inhibitor + %	Bond strength reduction (%) after aging		
				6 m	12 m	>12 m
Li et al. [9]	NA	NA	E&R			
			DMSO 1%	17.4^*∗*^		
			GD 5%	11.9^*∗*^		
			BAI 2.5 *μ*g/mL	10.1^*∗*^		
			Control	36.0		

Malaquias et al. [10]	Group: immediate/24 m Ambar®	50	E&R			24 m
	CHX 0.01%: 79.2/84.3		CHX 0.01%			16.7^*∗*^
	CHX 0.05%: 81.3/88.1		CHX 0.05%			17.0^*∗*^
	CHX 0.1%: 80.5/86.9		CHX 0.1%			10.0^*∗*^
	CHX 0.2%: 72.7/76.8		CHX 0.2%			10.4^*∗*^
	Control: 81/83.4		Control			40.2
	Group: immediate/24 m XP-Bond®		E&R			
	CHX 0.01%: 75.7/77.7		CHX 0.01%			33.8^*∗*^
	CHX 0.05%: 81/78.4		CHX 0.05%			32.1^*∗*^
	CHX 0.1%: 69.8/94.8		CHX 0.1%			29.1^*∗*^
	CHX 0.2%: 74/82.1		CHX 0.2%			29.3^*∗*^
	Control: 81.6/77.2		Control			53.3

Ou et al. [11]	Group: immediate/6 m/12 m	60	E&R			
	MMP8-I/90/70/75		MMP8-I	1.1^*∗*^	1.8^*∗*^	
	CHX 2%: 100/95/95		CHX 2%	0.7	5.3^*∗*^	
	Control: 95/80/90		Control	17.2	24.1	

El Gezawi et al. [12]	Group: immediate/6 m *µ*TBS	48				
	MDPB: 68/74		MDPB-SE	30.2		
	BAC: 68/72		BAC-PA	58.5		
	Control: 72/78		Control	64.3		
	4-point loading					
	MDPB: 68/70		MDPB-SE	31.6^*∗*^		
	BAC: 72/86		BAC-PA	62.9		
	Control: 78/84		Control	69.1		

Maravic et al. [13]	Group: immediate/12 m	36	E&R			
	ACR: 65/90		ACR 0.01%		14.4^*∗*^	
	Control: 67/82		Control		46.1	

Giacomini et al. [14]	Group: immediate/6 m Normal dentin	90	E&R			
	CHX 2%: 100/100		CHX 2%	41.8^*∗*^		
	E-64: 96/96		E-64 5 *μ*m	26.6^*∗*^		
	Control: 96/92		Control	22.3		
	Artificially carious dentin					
	CHX 2%: 100/100		CHX 2%	9.9		
	E-64: 96/96		E-64. 5 *μ*m	15.1		
	Control: 100/100		Control	13.4		
	Eroded dentin					
	CHX 2%: 100/100		CHX 2%	10.7^*∗*^		
	E-64: 100/100		E-64 5 *μ*m	8.4		
	Control/96/100		Control	12.7		

Daood et al. [15]	Group: immediate/12 m Adper™ Single Bond 2	60	E&R			
	CHX 2%: 64/72		CHX 2%	4^*∗*^	8^*∗*^	
	QAS 2%: 52/65		QAS 2%	−8.1^*∗*^	−3.6^*∗*^	
	QAS 5%: 75/64		QAS 5%	4.2^*∗*^	−6.0^*∗*^	
	QAS 10%: 72/67		QAS 10%	10.5	23.1	
	Control: 63/66		Control	14.5	29.5	
	Prime & Bond® NT™		E&R			
	CHX 2%: 80/55		CHX 2%	3.3	8.8^*∗*^	
	QAS 2%: 59/64		QAS 2%	−2.2	−4.4^*∗*^	
	QAS 5%: 74/74		QAS 5%	5.6^*∗*^	11.3^*∗*^	
	QAS 10%: 75/79		QAS10%	6.9^*∗*^	13.8^*∗*^	
	Control: 64/74		Control	12.2	20.9	

Venigalla et al. [16]	Group: immediate/6 m	80	E&R			
	RF WWB: 73/47		RF 0.1% WWB	2.5^*∗*^		
	CD WWB: 73/60		1 m CD WWB	5.6^*∗*^		
	PAC WWP: 67/47		6.5% PAC WWB	17^*∗*^		
	Control WWB: 87/60		Control WWB	24		
	RF EWB: 53/47		RF 0.1% EWB	0.6^*∗*^		
	CD EWB: 67/60		1 m CD EWB	4.7^*∗*^		
	PAC EWB: 60/53		6.5% PAC EWB	5.6^*∗*^		
	Control EWB: 67/47		Control EWB	10.2		

Carvalho et al. [17]	Group: immediate/6 m	30	E&R			
	Green tea: 36/68		Green tea 2%	−55.2^*∗*^		
	CHX 2%: 66/78		CHX 2%	1.3		
	Control: 81/68		Control	11.1		

Barcellos et al. [18]	NA	60	E&R			
			ZnOn 1 wt.%	3.1^*∗*^		
			Zn-Mt, 1 wt.%	46.1		
			Control	44.4		

Hass et al. [19]	NA	40	E&R Single Bond Plus®			18 m
			PAC 6.5wt%			11.9^*∗*^
			UVA-RF 0.1wt%			14.8^*∗*^
			GD 5wt%			22.9^*∗*^
			Control			64.8
			E&R Tetric N-Bond®			
			PAC 6.5wt%			5.5^*∗*^
			UVA-RF 0.1wt%			20.3^*∗*^
			GD 5wt%			32.2^*∗*^
			Control			62.2

Loguercio et al. [20]	Group: immediate/24 m Prime & Bond NT®	30	E&R			24 m
	MC 2%: 97/94		MC 2%			10.6^*∗*^
	CHX 2%: 85/94		CHX 2%			17.9^*∗*^
	Control: 80/78		Control			44.2
	Adper™ Single Bond 2		E&R			
	MC 2%: 98/96		MC 2%			10.9^*∗*^
	CHX 2%: 93/90		CHX 2%			13.9^*∗*^
	Control: 93/95		Control			30.1

Hass et al. [21]	Group: immediate/6 m	10	E&R			
	PAC 2%-PA: 84/82		PAC 2%-PA	−2.7^*∗*^		
	Control: 97/100		Control	47.2		

Tekçe et al. [22]	Group: immediate/12 m SB Universal®	50				
	BAC 1%: 58/75.5		BAC 1%		23	
	CHX 2%: 70.4/76		CHX 2%		8.9	
	EDTA 0.5 m: 66.1/73.4		EDTA 0.5 m		−0.9	
	SE: 60.6/78.3		SE control		3.6	
	E&R: 58.4/79		E&R control		13.1	
	All-Bond Universal®					
	BAC 1%: 70.2/71		BAC 1%		15.2	
	CHX 2%: 60.5/73.5		CHX 2%		19.4	
	EDTA 0.5 m/57/76		EDTA 0.5 m		17.6	
	SE: 78.6/90		SE control		21.6	
	E&R: 65.5/71		E&R control		12.0	

Abu Nawareg et al. [23]	Group: immediate/6 m/12 m	36	E&R			
	CHX 2%: 93.3/86.7/86.7		CHX 2%	3.5^*∗*^	5.9^*∗*^	
	CHX-MA 2%: 86.7/100/80		CHX-MA 2%	−5.1^*∗*^	−4.9^*∗*^	
	Control: 93.3/100/86.7		Control	22.9	33.3	

da Silva et al. [24]	Group: immediate/6 m/12 m Experimental adhesive	36	E&R			
	GAL 5 *μ*m: 77/77/59		GAL 5 *μ*m	13.3	17.5	
	BAT 5 *μ*m:71/71/84		BAT 5 *μ*m	10.9	15.3	
	GM1 5 *μ*m: 72/72/57		GM1 5 *μ*m	11.5	15.1	
	CHX 2%: 70/72/63		CHX 2%	12.5	13.9	
	Control: 79/75/83		Control	0.1	23.5	
	Single Bond 2: 68/74/84		Single Bond 2	5.7	20.3	

Montagner et al. [25]	NA	36	E&R			18 m
			CHX 2%			20.6
			NaOCl			25.7
			Control			44.6

Sabatini et al. [26]	NA	25	E&R			
			CHX 2%		1.3^*∗*^	
			BAC-PA 1%		53.2	
			BAC 0.5%		9.1^*∗*^	
			BAC 1%		28.3^*∗*^	
			Control		43.9	

Sabatini and Pashley [27]	NA	35	E&R			
			0.5% BAC	−2.6^*∗*^	1.6^*∗*^	
			1.0% BAC	4.5^*∗*^	−7.0^*∗*^	
			2.0% BAC	5.5^*∗*^	13.4^*∗*^	
			0.5% MBAC	−11.2^*∗*^	−26.4^*∗*^	
			1.0% MBAC	−5.4^*∗*^	−23.1^*∗*^	
			2.0% MBAC	5.6^*∗*^	1.0^*∗*^	
			Control	44.2	48.0	

André et al. [28]	NA	60	E&R			
			GD		5.8	
			GD-control		32.1^*∗*^	
			MDPB		−8.9	
			MDPB-control		19.2	
			0.2% CHX		2.5	
			0.2% CHX-control		13.5	

Manso et al. [29]	Group: immediate/6 m/15 m All-Bond 3®	48	E&R			15 m
	CHX 1% W: 73.5/51.6/58.3		CHX 1% water	−7.9		1.9
	Control W: 50/44.5/50		Control water	−11.9		7.4
	CHX 1% E: 75/55.8/65.3		CHX 1% ethanol	4.6		27.6
	Control E: 56.8/62.7/54.1		Control ethanol	5.1		25.3
	Excite®		E&R			
	CHX 1% W: 77.7/73.8/76		CHX 1% water	8.2		−2.1
	Control W: 78/81.5/69.1		Control water	15.0		8.1
	CHX 1% E: 62.1/47.2/59.2		CHX 1% ethanol	−7.7		7.2
	Control E: 56.7/41.2/76.6		Control ethanol	10.3		14.5

Ekambaram et al. [30]	Group: immediate/12 m Sound dentin	48	E&R			
	EWB + CHX: 93.7/75		EWB + CHX 2%		4.9^*∗*^	
	EWB control: 100/68.8		EWB control		21.2^*∗*^	
	WWB + CHX: 100/100		WWB + CHX		0.3^*∗*^	
	WWB control: 93.8/100		WWB control		27.6	
	Caries-affected dentin		E&R			
	EWB + CHX: 68.8/81.2		EWB + CHX		6.4^*∗*^	
	EWB control: 75.5/62.6		EWB control		14.4^*∗*^	
	WWB + CHX: 50/100		WWB + CHX		18.7^*∗*^	
	WWB control: 56.3/62.5		WWB control		60.9	

Sabatini and Patel [31]	Group: immediate/6 m/18 m OptiBond Solo Plus®	140	E&R			18 m
	2% CHX: 70/70/60		2% CHX	12.4		6.0
	BAC‐PA: 60/60/60		BAC‐PA	−4.7		−27.1
	0.25% BAC: 60/70/60		0.25% BAC	32.4		30.9
	0.5% BAC: 70/70/80		0.5% BAC	1.8		−95.0^*∗*^
	1.0% BAC: 70/70/100		1.0% BAC	−21.2^*∗*^		-46.3^*∗*^
	2.0% BAC: 80/70/70		2.0% BAC	15.7^*∗*^		−19.1
	Control: 70/70/80		Control	−3.5		−1.9
	All-Bond 3®		E&R			18 m
	2% CHX: 80/70/70		2% CHX	−13.8		15.5
	BAC‐PA: 90/80/50		BAC‐PA	−26.5		−9.6
	0.25% BAC: 70/70/60		0.25% BAC	11.1		−33.3
	0.5% BAC: 70/60/70		0.5% BAC	9.8		−41.0
	1.0% BAC: 90/80/90		1.0% BAC	−0.5		−22.2
	2.0% BAC: 90/80/70		2.0% BAC	10.7		−8.5
	Control: 80/70/70		Control	−20.9		−15

Pomacóndor-Hernández et al. [32]	NA	8	SE			
			CHX 2%	−8.1		
			Control	2.7		

Verma et al. [33]	NA	120	E&R Solobond M®			
			CHX 2%	−8.6^*∗*^		
			PAC 30%	6.8^*∗*^		
			Control	45.1		
			Tetric N Bond®			
			CHX 2%	0.9^*∗*^		
			PAC 30%	0.7^*∗*^		
			Control	36.2		

Tjäderhane et al. [34]	NA	20	DMSO 0.5 mm	−15.7^*∗*^	−36.4^*∗*^	
			Control	37.2	30.4	
			DMSO 0.5 mm	−12^*∗*^	−6.6^*∗*^	
			Control	22.2	42.0	

Sabatini et al. [35]	Group: immediate/6 m	25	E&R			
	CHX 2%: 70/60		CHX 2%	10.4^*∗*^		
	BAC-PA: 80/80		BAC 1% PA	18.4^*∗*^		
	BAC 0.5%: 70/80		BAC 0.5%-adhesive	−0.5^*∗*^		
	BAC 01%: 80/70		BAC 1%-adhesive	−4.9^*∗*^		
	Control: 70/60		Control	20.1		

Simoes et al. [36]	NA	36	E&R			
			CHX	28.5		
			Control	32.7		
			CHX + ethanol	21.4		
			Ethanol control	7.6		

Sabatini [37]	Group: immediate/6 m	120	E&R			
	CHX 2% + 0.2% CHX-adhesive: 70/50		CHX 2% + 0.2% CHX-adhesive	−4.2		
	0.2%-adhesive: 80/60		0.2% CHX-adhesive	5.2		
	Control: 90/70		Control	−14.3		
			SE			
	CHX 2% + 0.2% CHX-adhesive: 60/60		CHX 2% + 0.2% CHX-adhesive	−13.8		
	0.2% CHX-adhesive: 50/80		0.2% CHX-adhesive	−17.4		
	Control: 60/80		Control	−6.3		

Ali et al. [38]	NA	30	SE			
			2% CHX-ethanol	64.2^*∗*^		
			2% CHX-dH_2_O	−13.8^*∗*^		
			Control	36.5		

Leitune et al. [39]	NA	40	E&R			
			CHX 2%	−9.8^*∗*^		
			Control	10.9		

Cova et al. [40]	Group: immediate/6 m/12 m	60	E&R			
	RF 0.1%: 98/95/97		RF 0.1%	19.8^*∗*^	30.4^*∗*^	
	Control: 89/89/95		Control	41.0	52.6	

Mobarak [41]	NA	120	SE			24 m
			CHX 2%			ND 63.3 AD 52.1
			CHX 5%			ND 57.7 AD 28.8^*∗*^
			Control			ND 61.1 AD 54.1

Sadek et al. [42]	NA	42	E&R SB Multipurpose®			
			CHX 2% WWB			9 m: 9.5 18 m: 26.1
			Control			9 m: 15.5 18 m: 26.2
			Single Bond 2®			
			CHX 2% WWB			9 m: 10.4 18 m: 32.4
			Control			9 m: 18.9 18 m: 25.7
			Experimental			
			CHX 2% EWB			9 m: 4.7 18 m: 7.0
			Control			9 m: 3.0 18 m:3.3

Stanislawczuk et al. [43]	NA	42	E&R Prime & Bond NT®			24 m
			Control			53.5
			CHX			19.2^*∗*^
			CHX-PA			21.8^*∗*^
			E&R Adper Single Bond®			
			Control			46.9
			CHX			18.9^*∗*^
			CHX-PA			16.3^*∗*^

De Munck et al. [44]	Group: Immediate/6 m/12 m Scotchbond 1XT®	45	E&R			
	Control: 20/70/70		Control	42.9	49.5	
	CHX: 63/69/100		CHX	41.4	78.9	
	SB‐3CT: 33/100/100		SB‐3CT	76.5	93.6	
	Clearfil Protect Bond®		SE			
	Control: 0/60/54		Control	21	33.5	
	CHX: 0/63/67		CHX	33.1	48.3	
	SB-3CT: 6/32/82		SB-3CT	30.4	57.5	
	G-Bond®		SE			
	Control: 95/95/100		Control	52.1	66.2	
	CHX: 90/93/100		CHX	35.3	79.1	
	SB-3CT: 89/100/100		SB-3CT	62.3	60.8	

Ricci et al. [45]	Group: immediate/10–12 m/18–20 m	26	E&R		10–12 m	18–20 m
	CHX 2%: 100/100/100		CHX 2%		26.3	37.0^*∗*^
	Control: 75/87.5/100		Control		43.9	56.5

Breschi et al. [46]	Group: immediate/12 m	28	E&R			
	GAL/85/75		GAL 0.04%		26.5^*∗*^	
	Control: 65/76		Control		45.4	

Breschi et al. [47]	Group: immediate/24 m	48	E&R			24 m
	Control: 100/80		Control			67.2
	CHX 0.2%: 85/100		CHX 0.2%			16.8^*∗*^
	CHX 2%: 90/75		CHX 2%			30.8^*∗*^

Loguercio et al. [48]	Group: immediate/6 m Prime & Bond 2.1®	120	E&R			
	Control: 75/80.9		Control	33.4		
	0.002%: 77.6/71.7		CHX 0.002%	11		
	0.02%: 67.9/75		CHX 0.02%	−0.7^*∗*^		
	0.2%: 87.5/94.1		CHX 0.2%	11.3^*∗*^		
	2%: 75/94.4		CHX 2%	8.5^*∗*^		
	4%: 88.2/76		CHX 4%	21		
	Adper Single Bond®		E&R			
	Control: 81.2/69		Control	29		
	0.002%: 73.5/76.1		CHX 0.002%	11.6		
	0.02%: 46.4/75		CHX 0.02%	9.6^*∗*^		
	0.2%: 80/88		CHX 0.2%	−5.8^*∗*^		
	2%: 84.1/97.1		CHX 2%	12.7^*∗*^		
	4%: 77.6/70.2		CHX 4%	7.6		

Stanislawczuk et al. [49]	Group: immediate/6 m Prime & Bond NT®	42	E&R			
	Control: 67/75.8		Control	33.6		
	CHX 2%: 75/96.5		CHX 2%	−6.8^*∗*^		
	CHX 2%-PA: 83/72.4		CHX 2%-PA	16^*∗*^		
	Single Bond 2®		E&R			
	Control: 85/64.3		Control	25		
	CHX 2%: 96.3/100		CHX 2%	0^*∗*^		
	CHX 2%-PA: 87.6/76.9		CHX 2%-PA	4.6^*∗*^		

Zhou et al. [50]	Group: immediate/12 m	16	SE			
	0.05% CHX: 93.8/100		0.05% CHX		18.1	
	Control: 87.5/93.8		Control		18.7	
	0.1% CHX: 75/93.8		0.1% CHX		−0.8^*∗*^	
	Control: 93.8/100		Control		16.0	
	0.5% CHX: 87.5/100		0.5% CHX		5.9^*∗*^	
	Control: 93.8/100		Control		21.8	
	1% CHX/93.8/93.8		1% CHX		2.9^*∗*^	
	Control: 87.5/100		Control		15.6	

Breschi et al. [51]	Group: immediate/6 m/12 m Single Bond 1XT®	108	E&R			
	CHX 2%: 90/95/95		CHX 2%	11	24.6^*∗*^	
	CHX 0.2%: 100/95/100		CHX 0.2%	16.5	20.8^*∗*^	
	Control: 100/90/90		Control	38.0	54.2	
	XP-Bond®		E&R			
	CHX 2%: 100/90/85		CHX 2%	14.4	24.2^*∗*^	
	CHX 0.2%: 95/100/90		CHX 0.2%	13.1	30.8^*∗*^	
	Control: 100/95/85		Control	33.1	64.1	

**Table 2:** Factors associated with the risk of bias in different studies.

Study	Materials	Caries	Adhesive	Sample	Blinding	Random	Risk
Ou et al. [11]	Y	Y	NM	NM	NM	Y	Medium
Giacomini et al. [14]	Y	Y	NM	NM	NM	NM	High
Daood et al. [15]	Y	Y	NM	NM	NM	Y	Medium
Carvalho et al. [17]	NM	N	Y	NM	NM	Y	High
Abu Nawareg et al. [23]	Y	Y	NM	NM	NM	Y	Medium
Loguercio et al. [20]	Y	Y	Y	NM	NM	Y	Medium
Tekçe et al. [22]	Y	Y	NM	NM	NM	Y	Medium
Montagner et al. [25]	Y	Y	Y	NM	NM	Y	Medium
Ekambaram et al. [30]	NM	Y	NM	NM	NM	Y	High
Sabatini et al. [31]	Y	Y	NM	NM	NM	Y	Medium
Verma et al. [33]	NM	Y	NM	NM	NM	Y	High
Sabatini et al. [35]	Y	Y	NM	NM	NM	Y	Medium
Sabatini [37]	Y	Y	NM	NM	NM	Y	Medium
Leitune et al. [39]	Y	Y	NM	NM	NM	Y	Medium
Stanislawczuk et al. [43]	NM	Y	Y	NM	NM	NM	High
Sadek et al. [42]	Y	Y	NM	NM	NM	Y	Medium
Ricci et al. [45]	Y	*N*	Y	NM	NM	Y	Medium
Loguercio et al. [48]	Y	Y	Y	NM	NM	Y	Medium
Stanislawczuk et al. [49]	NM	Y	Y	NM	NM	NM	High
Breschi et al. et al. [51]	Y	Y	NM	NM	NM	Y	Medium
Loguercio et al. [48]	Y	Y	Y	NM	NM	Y	Medium

Total 21	16	19	8	0	0	18	

## Data Availability

The data used to support the findings of this study are available from the corresponding author upon request.
